# RNAi-based pest control: Production, application and the fate of dsRNA

**DOI:** 10.3389/fbioe.2022.1080576

**Published:** 2022-11-29

**Authors:** Li He, Yanna Huang, Xueming Tang

**Affiliations:** ^1^ School of Agriculture and Biology, Shanghai Jiao Tong University, Shanghai, China; ^2^ Key Laboratory of Urban Agriculture, Ministry of Agriculture and Rural Affairs, Shanghai, China; ^3^ Shanghai Yangtze River Delta Eco-Environmental Change and Management Observation and Research Station, Ministry of Science and Technology, Ministry of Education, Shanghai, China

**Keywords:** RNA pesticides, mass production, exogenous application, dsRNA fate, nanoparticles, synergistic effect, regulatory framework

## Abstract

The limitations of conventional pesticides have raised the demand for innovative and sustainable solutions for plant protection. RNA Interference (RNAi) triggered by dsRNA has evolved as a promising strategy to control insects in a species-specific manner. In this context, we review the methods for mass production of dsRNA, the approaches of exogenous application of dsRNA in the field, and the fate of dsRNA after application. Additionally, we describe the opportunities and challenges of using nanoparticles as dsRNA carriers to control insects. Furthermore, we provide future directions to improve pest management efficiency by utilizing the synergistic effects of multiple target genes. Meanwhile, the establishment of a standardized framework for assessment and regulatory consensus is critical to the commercialization of RNA pesticides.

## 1 Introduction

Insects cause up to 40% of the loss of crops worldwide every year and food security has always been the primary issue facing human survival and development. To meet the increasing demands of a growing world population, chemical pesticides have been widely used to reduce damages caused by pests and improve the quality and yield of products. However, the frequent use of pesticides has led to an increase in pesticide resistance and raised public concerns about its adverse effects on the environment and human health (Rank and Koch, 2021; Tudi et al., 2021). Therefore, it is necessary to explore innovative and sustainable approaches to protect crops.

RNA interference (RNAi), a highly conserved sequence-specific method of inhibiting a targeted gene’s expression, emerges as a practical technology to control insects in a species-specific manner. Although the transgenic maize SmartStax^®^ Pro that is engineered to express dsRNA targeting *Diabrotica virgifera virgifera Dvsnf7* was approved in Canada (2016) and the United States (2017) (Head et al., 2017), RNAi-based transgenic plants face great constraints due to the public concerns about the safety of the transgenic plants and the shortage of genetic transformation technology in some crops (Rank and Koch, 2021; Touzdjian Pinheiro Kohlrausch Tavora et al., 2022). Alternatively, RNAi-based non-transgenic products can be applied exogenously and are expected to reach global markets soon. In the context, we introduce the cost-effective method for mass production of dsRNA as well as the non-transgenic dsRNA delivery approaches. Also, we introduce what the exogenously applied dsRNA would experience before triggering insect RNAi responses.

## 2 dsRNA production

**FIGURE 1:**
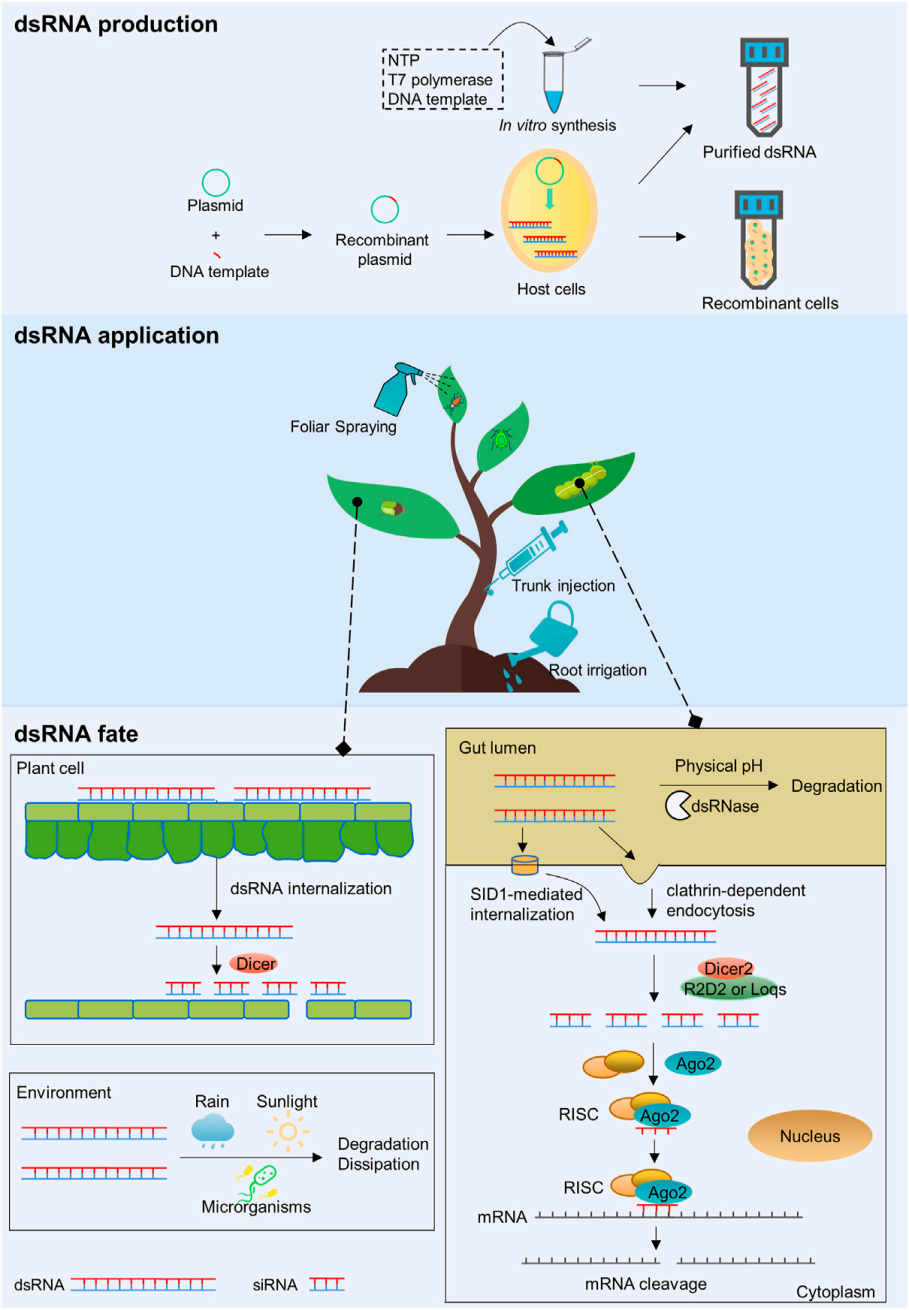
The schematic diagram of production, application and the fate of dsRNA.

Although field experimentation is still lacking, approximately 2–10 g of dsRNA per hectare is predicted to be needed for crop protection (Zotti et al., 2018). The usage of dsRNA for crop protection depends on the development of cost-effective methods for the mass production of dsRNA. Recently, *in vivo* production systems with engineered microorganisms as well as *in vitro* synthesis strategies with RNA polymerase allow large-scale dsRNA production (Table 1; Figure 1).

**TABLE 1:** The advantages and disadvantages of the dsRNA production system.

dsRNA production system	Application formulations	Advantages	Disadvantages
*E.coli*	Recombinant strains; purified dsRNA	Easy to genetically manipulate; Mature fermentation systems	May yield poor-quality dsRNA duplexes with errors; Cause environmental issues when applying the recombinant strains to the filed
Symbiotic bacteria	Strains	Use the host pests to express dsRNA; Can be used directly without dsRNA isolation; Can be spread to the colony horizontally and vertically	Hard to isolate symbiotic bacteria; Hard to stably re-colonize the symbiotic bacteria; Hard to obtain RNaseIII-deficient dsRNA-expressing symbiotic strains
Yeast	Strains	Non-toxic to humans; Can be used directly without dsRNA isolation; Easy to genetically manipulate; Mature fermentation systems	May yield poor-quality dsRNA duplexes with errors
Bacteriophage	Purified dsRNA	Do not need error-prone hybridization processes	Need multiple expression cassettes/elements to express dsRNA
Cell-free production platform	Purified dsRNA	High purity	Need multiple robust enzymes

### 2.1 *Escherichia coli*

To date, the majority of studies have used *E. coli* strains, especially the RNaseIII-deficient *E. coli* HT115/DE3, to produce dsRNA under the control of an inducible T7 promoter. Initially, the recombinant *E. coli* was engineered for dsRNA production and fed to *Spodoptera exigua* to evoke RNAi (Tian et al., 2009). Subsequently, ingestion of either live or heat-killed *E. coli* strains expressing dsRNA has been proven to successfully trigger RNAi responses in many insect species such as *Bactrocera dorsalis* (Li et al., 2011), *Aedes aegypti* (Whyard et al., 2015), *Leptinotarsa decemlineata* (Xu et al., 2019), *Maruca vitrata* (Al Baki et al., 2020), and *Nylanderia fulva* (Meng et al., 2020).

The yields of dsRNA are correlated with the expression plasmid and *E. coli* strains. Vectors used to express dsRNA contain either single or dual T7 promoters and the construct harboring a single T7 promoter (such as pGEM-T and pET28a/22b) appears to produce higher yields of dsRNA than that harboring dual T7 promoters (such as pL4440) (Yin et al., 2009; Ma et al., 2020). It is likely the consumed energy during transcription may differ between single and dual T7 promoters. In addition, RNA transcribed from a single T7 promoter contains two inverted complementary strands flanked by a loop that may be readily annealed; while dual T7 promoters allow bi-directional transcription of the insert and the transcribed ssRNA may be easily degraded by the RNA endonucleases before forming dsRNA. Notably, the yield of dsRNA is also affected by the host strains. For example, dsRNA produced by the RNaseIII-deficient strains of M-JM109 and M-JM109lacY is higher than that produced by HT115 (DE3) strains (Yin et al., 2009). Interestingly, pET28-BL21 (DE3) RNase III- system yields higher quantities of dsRNA than the pET28-HT115 (DE3) system, while the L4440-BL21 (DE3) RNase III-system has lower dsRNA expression efficiency than L4440-HT115 (DE3) (Ma et al., 2020). The inconsistent dsRNA expression efficiency may be due to the sub-optimal conditions for dsRNA production and optimizing the nutrition and fermentation approaches will improve dsRNA synthesis efficiency (Guan et al., 2021; Nwokeoji et al., 2022).

Although IPTG is a popular reagent for the induction of dsRNA expression in the vector systems harboring T7 promoter, the cost and the toxicity of IPTG should be taken into consideration (Dvorak et al., 2015). Currently, lactose and skimmed milk have been used to substitute IPTG for dsRNA production (Papic et al., 2018; Khani and Bagheri, 2020; Delgado-Martin and Velasco, 2021; Nwokeoji et al., 2022). These cheap and natural materials will have great potential in the scalable production of dsRNA. In addition, the utilization of a constitutive expression system can be another approach to express dsRNA without the addition of inducers (Delgado-Martin and Velasco, 2021).

### 2.2 Symbiotic bacteria

Symbiotic bacteria wildly exist in the gut of insects and utilizing symbiotic bacteria to express dsRNA is a promising strategy. Whitten, et al. (2016) genetically engineered the symbiotic bacteria *R. rhodnii* and *BFo2* that were isolated from the gut of *Rhodnius prolixus* and *Frankliniella occidentalis* respectively to generate RNase III–deficient, dsRNA-expressing strains. Upon ingestion, the engineered strains could successfully colonize and persist in the insect, allowing the constitutive synthesis of dsRNA to evoke effective RNAi responses in insects (Whitten et al., 2016). In *Apis mellifera*, the engineered gut bacterium *Snodgrassella alvi* could stably recolonize bees and produce dsRNA to protect bees from mites and viral challenges (Leonard et al., 2020). Interestingly, the symbiotic bacteria in *R. rhodnii* can be horizontally spread to other individuals *via* the feces (Whitten et al., 2016), while the symbiotic bacteria *Serratia* isolated from *Anopheles stephensi* ovaries can be sexually transmitted from males to females and spread to the offspring from one generation to the next (Wang et al., 2017). Horizontal and vertical spread of symbiotic bacteria enable efficient dissemination of dsRNA-producing strains throughout insect populations, thereby enhancing symbiotic-mediated RNAi persistence and efficiency. Notably, it would be challenging to isolate and stably re-colonize the appropriate symbiotic bacteria in insects. In addition, there may be technical bottlenecks in generating RNaseIII-deficient dsRNA-expressing symbiotic strains.

### 2.3 Yeast

*Saccharomyces cerevisiae* (baker’s yeast) is non-toxic to humans and widely used as a dietary supplement. Its ease of genetic manipulation and lack of RNAi machinery make it a novel and ideal system to express and accumulate dsRNA (Drinnenberg et al., 2009; Stewart et al., 2020). Several studies have confirmed the feasibility of using yeast pesticides to control insects. For example, *Drosophila suzukii* that fed with *S. cerevisiae* expressing dsRNA targeting *D. suzukii* yTub23C showed a significant reduction in target gene expression, locomotor activity, survivorship as well as reproductive fitness (Murphy et al., 2016). After feeding on yeast strains expressing dsRNA, both larvae and adults of mosquitoes experienced decreased expression of the target genes and severe neurological defects and death (Hapairai et al., 2017; Mysore et al., 2017; Mysore et al., 2019; Hapairai et al., 2020; Mysore et al., 2020). Interestingly, the dried-inactivated and live yeast formulations have the same larvicidal activities (Hapairai et al., 2017; Mysore et al., 2017), and the dried yeast formulations can be distributed worldwide and alleviate the public’s safety concerns. In addition, the mosquito larvae fed with the stable yeast transformants exhibit similar mortality rates to that fed with the transient yeast transformants (Hapairai et al., 2017; Mysore et al., 2019). Integration of shRNA expression cassettes into the yeast genome could eliminate the use of plasmids with antibiotic resistance markers and reduce the potential risk for horizontal transfer of shRNA expression cassettes. Notably, the shRNA expression is induced under the control of the galactose-inducible *GAL1* promoter in the current stable yeast transformants. However, it is impractical for large-scale industrial fermentation to use galactose as an inducer of gene expression because galactose is more expensive than glucose and the *GAL1* promoter cannot be induced after the carbon source is shifted from glucose to galactose under anaerobic conditions (van den Brink et al., 2009). There is a need to select and assess the promoters that are more readily used in industrial-sized cultures.

### 2.4 Bacteriophage

Bacteria and yeast cells can be used to produce dsRNA in large quantities, which rely on the processes of DNA transcription and post-transcriptionally ssRNA hybridization. However, annealing of the two complementary ssRNA molecules might yield poor-quality dsRNA duplexes with errors. The bacteriophage phi6 is a dsRNA virus that utilizes the RNA-dependent RNA polymerase (RdRP) to generate dsRNA from an ssRNA template, which would be an excellent tool to eliminate error-prone hybridization. phi6 genome contains three segments termed “S” (2948 bp), “M” (4063 bp) and “L” (6374 bp). Simultaneous introduction of all three segments and their corresponding packaging signal into *Pseudomonas syringae* cells enables the synthesis of dsRNA molecules of L, M, and S. When replacing the M- and S- segments with *Tobacco mosaic virus* (TMV) sequences, Niehl, et al. (2018) successfully synthesized dsRNA of TMV that could inhibit the transmission of TMV virus in infected *Nicotiana benthamiana* plants (Niehl et al., 2018). Notably, the L-segment is indispensable in the dsRNA production system (Frilander et al., 1995). dsRNAs produced in the *P. syringae* cells inevitably contain L-segment dsRNA molecules. It is critical to use appropriate methods to quantify the produced dsRNA of the target genes, and the potential off-target effect caused by the L-segment should not be ignored as well. Furthermore, the lack of an M-segment might fail to yield the expected dsRNA (Aalto et al., 2007; Niehl et al., 2018). Incorporation of both M-and S-segment into a dsRNA production system would promote the stable production of dsRNA and the replacement of the M- and S-segment with different sequences allows for the simultaneous synthesis of dsRNAs for different target genes. In addition, the length of the target genes mimics the size of the natural phi6 M- and S-segment, whether the phi6-based dsRNA production system could be used to produce dsRNA with variable lengths needs further investigation.

### 2.5 *In vitro* dsRNA production

*In vitro* transcription kits utilizing purified RNA polymerases and nucleotides have been widely used in laboratory experiments, but the high cost (∼$700 per mg dsRNA) limits the large-scale application in the field. GreenLight Biosciences has developed a large-scale cell-free production platform that uses endogenous cellular RNA to synthesize the desired dsRNA. Briefly, the endogenous RNA was depolymerized into nucleoside monophosphates (NMPs) with nucleases and then phosphorylated with kinases to form nucleotide triphosphates (NTPs). These NTPs were then polymerized into the target dsRNA using the corresponding DNA template and RNA polymerases (Cunningham et al., 2020). Compared to fermentations that cost $1 to produce 1 g of dsRNA, this cell-free platform can produce 1 g of dsRNA for as little as $0.50, making it highly competitive in the market (http://www.globalengage.co.uk/pgc/docs/PosterMaxwell.pdf). Notably, a combination of different nucleases may be required to depolymerize the various types of endogenous cellular RNA including ssRNA and dsRNA. Also, the kinases and RNA polymerase should be thermostable when heating the mixture of cell lysates to inactivate nucleases and other endogenous enzymes.

## 3 Exogenous application of dsRNA in the field

After dsRNA production, proper approaches are needed to deliver dsRNA into the target organisms. Currently, three methods show great potential in dsRNA application in the field, including foliar spraying, root irrigation, and trunk injection (Figure 1).

### 3.1 Foliar spraying

Foliar spraying of dsRNA is an efficient method to control pests feeding/growing on stems, foliage, or fruits. In *Henosepilachna vigintioctopunctata*, spraying *E. coli* expressed dsRNA targeting the *ecdysone receptor* (*EcR*) onto the foliage of greenhouse-growing potato plants would inhibit larval-pupal transition and reduce leaf consumption (Wu et al., 2021). Similarly, foliar spraying dsRNA has been shown to protect potato plants from Colorado potato beetle larvae, and Ledprona targeting proteasome subunit beta 5 is under registration at the United States Environmental Protection Agency (San Miguel and Scott, 2016; Mehlhorn et al., 2020; Rodrigues et al., 2021).

To effectively control piercing-sucking insects as well as those hiding in fruits, stems and the back of leaves, the sprayed dsRNA is required to be internalization and spread by the plant cells, and several studies have confirmed the systemic spread of dsRNA. For example, the fluorescent-labeled dsRNA sprayed onto the barley leaves can be detected in xylem, phloem parenchyma cells, companion cells, mesophyll cells, trichomes and stomata cells *via* the plant vascular system (Koch et al., 2016). With aphid stylectomy, Biedenkopf, et al. (2020) visualized the phloem-mediated transfer of sprayed-dsRNA in the distal, non-sprayed barley leaves (Biedenkopf et al., 2020). The *Zucchini yellow mosaic virus* derived dsRNA could be detectable in non-sprayed tomato leaves, aphids (*Myzus persicae*) and whiteflies (*Trialeurodes vaporariorum*) 14 days post foliar spraying (Gogoi et al., 2017). Notably, long-distance spreading may result in dsRNA dilution and it is needed to figure out how much and how often the dsRNA needs to be sprayed to control pests efficiently.

### 3.2 Root irrigation

dsRNA can be absorbed by plants *via* root irrigation and transmitted to insects that feed on the treated plants, which offers an alternative method for pest management. For example, dsRNA targeting arginine kinase showed persistence in the citrus trees (2.5 m tall) for 57 days after root drench (2 g dsRNA/15 L water) and could be detectable in the psyllids and leafhoppers for 5–8 days after ingesting the treated plants (Hunter et al., 2012). When Asian corn borer (*Ostrinia furnacalis*) fed on maize seedlings that were irrigated with solutions containing dsRNA of *Kunitz-type trypsin inhibitors*, the expression level of the target genes was significantly decreased and its mortality rate was significantly increased. Similarly, soaking rice roots in a solution containing dsRNA targeting *carboxylesterase* (*Ces*) and *CYP18A1* enhanced rice resistance to the brown planthopper (*Nilaparvata lugens*), respectively (Li H. et al., 2015). Up to 80% of mortality rate was observed in *Tuta absoluta* feeding on the tomato leaves when the plant roots were immersed into *ryanodine receptors* (*RyRs*), *acetylcholinesterase* (*AChE*), and *nicotinic acetylcholine alpha 6* (*nAChRs*) dsRNA solutions (Majidiani et al., 2019). Simultaneous RNAi of *vestigial* (*vg*) and *Ultrabithorax* (*Ubx*) *via* root applications resulted in 32.2% wing aberration rates in *M. persicae* (Zhang et al., 2022).

### 3.3 Trunk injection

Trunk injection utilizes the tree’s vascular system to deliver injected pesticides to the canopy and fruit. The technique can protect dsRNA from degradation caused by UV exposure or being washed away, making it a promising approach to protect horticultural trees. In apple trees, dsRNA injected into the trunk could be detected in leaves for over 84 days, and the peak dsRNA concentrations in leaves were as high as 8 ng/1 g leaf tissue (Wise et al., 2022). However, it is unclear how effective the method is for controlling insects, and more research is needed.

## 4 The fate of dsRNA post application

The exogenously applied dsRNA would be exposed to the environment, absorbed by the plant cells, and ingested by the insects. Upon ingestion, dsRNA has to survive the harsh environment of the digestive tract and enter cells. The internalized dsRNA is then diced siRNA to trigger the RNAi machinery (Figure 1).

### 4.1 dsRNA in the environment

The sprayed dsRNA is not stable in the environment. UV light is known to degrade nucleic acids and a visible dsRNA degradation could be observed on the agarose gel after dsRNA was exposed to UV irradiation for 30 min (San Miguel and Scott, 2016). Similarly, Li H et al. (2015) discovered that dsRNA degraded gradually under continuous UV irradiation as well as sunlight (Li H. et al., 2015). The rain and dew would lead to dsRNA dissipation. In *Arabidopsis,* the leaves were rinsed with water 24 h post foliar spraying of Cy3-labeled CMV2b-dsRNA and most of the sprayed-dsRNA could be readily washed away as determined by confocal microscopy (Mitter et al., 2017). Surprisingly, San Miguel and Scott (2016) showed that whether rinsing the potato leaves after spraying actin-dsRNA would not affect the weight gain and mortality rate in *L. decemlineata*, suggesting that the strong adhesion characteristic of dsRNA onto the leaves (San Miguel and Scott, 2016). The contradictory results may be due to the different detection/quantification methods as well as the paucity of data, and more research is needed.

dsRNA in the soil would be rapidly degraded as well. DvSnf7-dsRNA degraded with a half-life of 15–28 h after being applied to the three representative agricultural soils including silt loam, loamy sand and clay loam soils, and the degradation rate was independent of the amount of dsRNA applied to the soils (Dubelman et al., 2014). A similar dissipation pattern was also observed when DvSnf7-dsRNA was incorporated in tropical soils from Brazil (Joaquim et al., 2019). Notably, the soil particles as well as the soil microorganisms would account for dsRNA dissipation in the soils (Parker et al., 2019). However, how the dsRNA dissipation affects RNAi efficiency needs further investigation.

The plant foliar cuticle and cell wall would act as barriers to the efficient uptake of dsRNA. When the adaxial surface of *Amaranthus palmeri* leaves was sprayed with Cy3-labeled siRNA, most of them were associated with the cuticle 4 h post application. Cell wall pore size would prevent nucleic acid uptake in BY-2 suspension cells, where 90 bp DNA is more difficult to be internalized by flag22-stimulated endosomes than 21 and 50 bp DNAs. Abrasion with microparticles or high-pressure spraying, abaxial stomatal flooding, and surfactant utilization have been shown to improve dsRNA penetration through the barriers and achieve robust RNAi phenotype (Bennett et al., 2020).

### 4.2 dsRNA in the plant cells

The absorbed dsRNA can be spread in the plant cells. For example, the fluorescent-labeled dsRNA sprayed onto the barley leaves could be detected in xylem, phloem parenchyma cells, companion cells, mesophyll cells, trichomes and stomata cells *via* the plant vascular system (Koch et al., 2016). With aphid stylectomy, Biedenkopf, et al. (2020) could visualize the sprayed-dsRNA in the non-sprayed barley leaves (Biedenkopf et al., 2020). The systemic spread of dsRNA provides a possibility to effectively control the insects feeding on stems, leaves or fruits.

dsRNA absorbed by the plants will be processed into siRNA *via* the plant’s intrinsic RNAi machinery. Silencing the plant *Dicer-like* enables the accumulation of long dsRNAs, resulting in an enhanced plant-mediated RNAi efficiency in *Helicoverpa armigera* and *Manduca sexta* (Mao et al., 2007; Kumar et al., 2012). Utilization of RNAi-deficiency plants seems to be a selective method to increase the pest control efficiency of dsRNA molecules. However, whether plants with decreased Dicer activity would be susceptible to viral pathogens and developmentally defective should be taken into consideration and needs further investigation.

### 4.3 dsRNA in the digestive system and hemolymph

Insect can ingest the exogenously applied dsRNA *via* feeding behavior or epidermal penetration. Upon ingestion, dsRNA has to survive the harsh environment in the digestive system and hemolymph before entering the cells to evoke RNAi machinery. In response to a feeding stimulus, the gut cells secrete a peritrophic matrix (PM), through which ingested dsRNA must pass before being taken up by intestinal epithelial cells. However, the presence of negatively charged proteoglycans in the PM would hinder the free transport of dsRNA through the PM because of the negatively charged phosphate backbone of dsRNA (Kunte et al., 2020). dsRNase in the gut fluid and hemolymph could degrade dsRNA and inhibition of dsRNase activity has been shown to enhance dsRNA stability and thus improve RNAi efficiency (Supplementary Table S1). For example, knockout of *Spodoptera litura dsRNase1 and dsRNase2* simultaneously resulted in a 96% decrease in dsRNA-degrading activity and decreased the target gene mRNA expression level by 23% (Peng et al., 2021). After silencing *Cylas puncticollis CpdsRNase3*, dsRNA stability in midgut juices was significantly prolonged and dsSnf7-feeding induced mortality was increased by 30% (Prentice et al., 2019). dsRNase activity varies in different developmental stages and different species, resulting in different RNAi responses. For example, the nuclease activity in *S. exigua* gut juice was relatively lower at the younger stages than that at the older stages, while the mortality was higher in the younger larvae than that in the older larvae after oral treatment with *dsSeCHY2*-expressing bacteria (Vatanparast and Kim, 2017). dsRNA was degraded more rapidly in gut extracts of RNAi-insensitive pea aphid (*Acyrthosiphon pisum*) than that of RNAi-sensitive red flour beetle (*Tribolium castaneum*) (Cao et al., 2018). Notably, the body size and weight of insect species have big differences and should be taken into consideration.

The physiological pH may affect dsRNA stability by influencing dsRNase activity. In *L. migratoria*, LmdsRNase1 that is mainly expressed in hemolymph could degrade dsRNA efficiently at an optimal pH of 5.0 but showed no degrading activity at the physiological pH 7.0 of hemolymph, resulting in high RNAi efficiency after dsRNA injection; whereas gut-specific LmdsRNase2 exhibited degrading activity at pH from 6.0 to 10.0 and could effectively digest dsRNA at the physiological pH of midgut juice (pH 6.8), leading to a very low RNAi efficiency after feeding of dsRNA (Song et al., 2017; Song et al., 2019). Interestingly, the type of ingested food may alter the dsRNase activity or pH in the gut (Peng et al., 2020). It would be promising to add additives to change the enzymatic activity or pH to enhance RNAi efficiency.

### 4.4 dsRNA cellular uptake in insects

dsRNA cellular uptake is an indispensable step to generate RNAi responses and two pathways have been identified to play roles in internalizing dsRNA from gut lumen: 1) Systemic RNA Interference Deficient protein 1 (SID1)-mediated uptake pathway and 2) clathrin-dependent endocytic pathway. Regarding the SID1-mediated dsRNA uptake, SID1 orthologues (also known as Sil) have been identified in many insects except Diptera (Horn et al., 2022). *A. mellifera* administrated with dsRNA showed a significant increase in *AmSid1* expression level (Aronstein et al., 2006) and knockdown of *Sil* led to a decreased RNAi efficiency in insects such as *L. decemlineata*, *N. lugens* and *D. virgifera virgifera*, suggesting the functional role of SID1 in dsRNA internalization (Xu et al., 2013; Miyata et al., 2014; Cappelle et al., 2016). However, suppression of *Sil* failed to abolish RNAi responses in insect species such as *P. xylostella*, *T. castaneum*, *Schistocerca gregaria*, and *Locusta migratoria* (Tomoyasu et al., 2008; Luo et al., 2012; Wang et al., 2014; Wynant et al., 2014). An alternative pathway would be responsible for dsRNA uptake in these species.

Clathrin-dependent endocytosis associated with dsRNA uptake in insects is first described in *Drosophila* S2 cells which lack a *sid1* homologous sequence (Saleh et al., 2006; Ulvila et al., 2006). In *T. castaneum*, RNAi response of *TcLgl* was significantly impaired when utilization of endocytosis inhibitors or suppression of genes encoding proteins involved in clathrin-dependent endocytosis, indicating the role of clathrin-mediated endocytosis in dsRNA cellular uptake processes (Xiao et al., 2015). With a similar approach, clathrin-dependent endocytosis has been identified for dsRNA internalization in other insects such as *L. decemlineata*, *B. dorsalis*, *D. virgifera virgifera* and *A. pisum* (Li X. et al., 2015; Cappelle et al., 2016; Pinheiro et al., 2018; Ye et al., 2021).

It is noteworthy that both SID1 and endocytosis pathways would be involved in dsRNA uptake in the same species such as the case in *L. decemlineata* (Cappelle et al., 2016). However, the synergetic effect has only been confirmed in limited species and it remains unknown whether the two pathways function individually or in tandem. In *Spodoptera frugiperda* overexpressing *CeSid1*, RNAi efficiency was enhanced in ovary-derived Sf9 cells and Verson’s gland tissues, but not improved in midgut-derived Sf17 cells and midgut tissues (Chen et al., 2021). The uptake mechanisms seem to differ in different tissues and more investigations are needed.

Long dsRNA seems to be more efficient to be internalization than short dsRNA molecules. In *D. virgifera virgifera*, the 240 bp Cy3-dsRNAs could be observed in midgut cells while the 21 bp Cy3-siRNAs were barely detectable (Bolognesi et al., 2012). In *Drosophila* S2 cells, transfection reagents were needed to aid the efficient cellular uptake of siRNA (Saleh et al., 2006). It should be noted that long dsRNA will increase the chances of off-target and non-target effects. Considering the diversity of organisms present in and around a given agroecosystem that are potentially exposed to the applied dsRNA, bioinformatic-based analysis would be helpful to minimize the potential environmental risk.

### 4.5 dsRNA processing in insects

Upon dsRNA uptake, the type III ribonuclease Dicer 2 (Dcr 2) cleaves the dsRNA into siRNA that is approximately 21 bp in length with 2 nucleotides overhanging at each 3’ end (Elbashir et al., 2001; Santos et al., 2019). The dsRNA binding protein R2D2 then binds to siRNA duplex and Dcr 2, allowing the loading of the siRNA duplex to Argonaute 2 (Ago 2) of the RNA-induced silencing complex (RISC) (Tomari et al., 2004). Within RISC, siRNA is unwound and one of the siRNA strands (“passenger” strand) is degraded; while the other strand (“guide” strand) is retained and directs the RISC to the complementary mRNA, resulting in the cleavage and degradation of target gene expression (Rand et al., 2004; Rand et al., 2005).

dsRNA treatment can rapidly and transiently increase the expression of core siRNA enzyme (e.g., Ago 2, Dcr 2), and *Ago 2* expression is independent of the Dcr 2 activity (Rubio et al., 2018; Cooper et al., 2019; Montanes et al., 2021). The expression of the core siRNA enzymes seems to be highly correlated with RNAi efficiency. Suppression of Dcr 2 or Ago2 would limit RNAi efficiency (Wynant et al., 2012; Li Z. et al., 2015; Velez et al., 2016; Yoon et al., 2016; Rubio et al., 2018); whereas overexpression of Dcr 2 and Ago 2 can lead to an enhanced RNAi efficiency in *D. melanogaster* and *Bombyx mori*, respectively (Dietzl et al., 2007; Li Z. et al., 2015). In RNAi-sensitive species such as *L. decemlineata* and *T. castaneum*, both two copies of Ago 2 genes were involved in dsRNA-triggered RNAi (Tomoyasu et al., 2008; Yoon et al., 2016). It is reasonable to suspect that the extra copy of the Ago 2 is responsible for the robust RNAi efficiency. Notably, gene duplications or deletions of Dcr 2 and Ago 2 have been identified in a variety of insect species (Dowling et al., 2016). However, more investigations are needed to determine the relationships between the copy number of siRNA pathway genes and the different RNAi sensitivity in insects.

The processing of dsRNA to siRNA is variable in different insects. The dsRNA in *L. decemlineata* tissues and cell lines could be efficiently processed into siRNA, while siRNA was undetectable in total RNA isolated from *Heliothis virescens* tissues and cell lines (Shukla et al., 2016). All tested Coleoptera exhibited efficient cleavage of injected or fed dsRNA to siRNA, whereas dsRNA processing into siRNA was less efficient in Hemiptera, Orthoptera, Diptera and Lepidoptera than that in Coleoptera (Singh et al., 2017). It is likely that the variations in the structure and activity of Dicer contribute to the different efficiency of dsRNA processing into siRNA in different insects. Notably, Loquacious-PD (Loqs-PD) isoform, a dsRNA-binding protein, could facilitate siRNA production by interacting with and modulating the ATP-dependent conformational changes of the helicase domain of Dicer-2 (Fukunaga et al., 2012; Sinha et al., 2015; Trettin et al., 2017; Fukunaga, 2018; Su et al., 2022). In addition, Staufen C (StauC) is also involved in processing dsRNA into siRNA (Yoon et al., 2018), and overexpression of StauC in *D. melanogaster* Kc cells could restore the loss-of-the function of Loqs-PD (Kim et al., 2021). Interestingly, StauC homologs have only been identified in RNAi-sensitive Coleoptera insects, suggesting a correlation between the presence of StauC and high RNAi efficiency.

## 5 Nanoparticle: An efficient dsRNA carrier

dsRNA would experience hostile environments before triggering RNAi responses and various types of nanoparticles have been proven as efficient dsRNA carriers that can be used to improve RNAi efficiency by enhancing dsRNA stability and dsRNA uptake (Pugsley et al., 2021; Silver et al., 2021; Yan et al., 2021). For example, the layered double hydroxide (LDH) nanocarrier termed “Bioclay” can protect the dsRNA from degradation by the UV irradiation, improve dsRNA adhesion to leaf surfaces, and enhance cellular uptake and spread of dsRNA, resulting in sustained release of dsRNA and extended protection period (Mitter et al., 2017; Jain et al., 2022). Incorporation of the shaped poly (2-(dimethylamino) ethyl acrylate into dsRNA would increase the lifetime of dsRNA in soil up to 3 weeks (Whitfield et al., 2018). During feeding, the cationic nanoparticles shielded the negatively charged dsRNA and promote the efficiency of transporting dsRNA through the peritrophic matrix (Kunte et al., 2020). When incubation dsRNA with gut juice of *S. exigua*, dsRNA associated with guanidine-containing polymers was persistence for up to 30 h, while the naked dsRNA was completely degraded within 1 h (Christiaens et al., 2018). Notably, several issues should be taken into consideration when utilization of nanoparticle-dsRNA delivery system for RNAi-based pest management, First, the raw materials used to synthesis nanoparticles should be cheap, non-toxic, and environmentally friendly. Second, nanoparticles should carry cationic group to bind to the negatively charged dsRNA phosphate groups. At the same time, dsRNA could be dissociated from the nanopaticles in the cells, allowing dsRNA to be processed into siRNA by Dicer 2 (Kunte et al., 2020). Third, some nanoparticles may clog pores and barriers in the apoplastic stream, resulting in reduced nutrient uptake, inhibited photosynthetic process and damaged DNA structures in plants (Tripathi et al., 2017). The impact of nanoparticles in the environment needs to be evaluated before they can be safely used on crops.

## 6 Future perspective

Given that RNAi has shown great potentials in controlling pests, global scientists, enterprises and government regulatory agencies need to work together to accelerate the commercialization of dsRNA insecticides. It will be obvious that the development of dsRNA pesticides to improve the control efficiency of target pests is of great importance in the commercialization of dsRNA pesticides. Also, adequate risk assessment is required to minimize off-target risks for non-target organisms and develop handling recommendations in the field. In addition, a regulatory framework is needed to direct the development of dsRNA pesticides.

### 6.1 Synergistic effects

dsRNAs targeting multiple genes have shown potential for synergistic effects. In *Agrilus planipennis*, larvae fed with dsIAP and dsCOP sequentially showed a higher mortality (55%) than that with only dsIAP (33%) or dsCOP (24%) (Rodrigues et al., 2017). Simultaneous ingestion of both dsRNAs at low concentrations (1 μg/μL) caused up to 90% mortality, while dsRNA treatment alone showed similar mortality but at much higher concentrations (10 μg/μl) (Rodrigues et al., 2018). *Chilo suppresallis* larvae fed with the mixture of dsCYP15C1 and dsC-factor showed approximately 40% higher mortality than those fed with either dsCYP15C1or dsC-factor alone, while dsRNA complexed with DMAEMA polycationic nanomaterial resulted in at least 10% higher mortality than the naked dsRNAs (Niu et al., 2022). Association nanoparticles with dsRNA targeting multiple genes will further increase the synergistic effects. Interestingly, *T. castaneum* larvae simultaneously fed with two dsRNAs complexed with BACPs showed 20% and 30% higher mortality than those fed with dsBiP/BACPs and dsArmet/BACPs, respectively (Avila et al., 2018), while larvae injected with combinations of two dsRNAs showed no synergistic effects (Ulrich et al., 2015). Silencing multiple genes in *Aphis glycines* seemed to have lower mortality compared to silencing two genes (Yan et al., 2019). The failure to show a synergistic effect may due to overloading of the RNAi machinery and it is needed to determine the optimal values for the number of target genes and dsRNA concentration ranges. Also, the dsRNA delivery method may affect synergistic effects and more investigation is needed.

### 6.2 dsRNA pesticides risk assessment

Insects can be resistant to almost all conventional chemical insecticides. However, the mechanism of insect resistance to dsRNA insecticides is different. The mismatch between dsRNA and target mRNA sequences caused by gene mutations or polymorphisms can drive the evolution of resistance (Yu et al., 2016). Insects can become resistance to RNAi by preventing cellular uptake of dsRNA as well. In *D. virgifera virgifera*, the Cy3-labeled-DvSnf7 dsRNA could be observed inside the midgut cells of the RNAi-susceptible population but not the RNAi-resistance colony (Khajuria et al., 2018). In *B. dorsalis*, genes required for dsRNA internalization were suppressed in RNAi refractory flies and the RNAi refractoriness was disrupted when the endocytic capacity was increased by improving F-actin polymerization (Li X. et al., 2015). In addition, down-regulation or mutation of genes involved in RNAi machinery genes is a potential mechanism for resistance development. In *L. decemlineata*, Staufen C, a dsRNA-binding protein that is required for dsRNA processing, was expressed at lower levels in RNAi-resistant cells than in RNAi-susceptible cells (Yoon et al., 2018). Interestingly, RNAi efficiencies differed among three different field populations of *D. virgifera virgifera* even though there are no sequence differences in the target gene region (Chu et al., 2014), suggesting that the inherent physiological and genetic variation will lead to the development of resistance. Notably, the resistance caused by target gene mutation can be easily mitigated by utilizing a dsRNA that targets a different region or a different gene, which is also one of the unique advantages of RNA insecticides in pest resistance management.

RNA molecules are natural components of food and consumed by humans and other vertebrates and invertebrates. However, in order to avoid the potential risks of RNAi products, researchers need to rule out that dsRNA may impose risks on non-target organisms in a sequence-specific manner when designing exogenous dsRNA insecticides. Usually, bioinformatics-based analysis can help eliminate out of target effects by constantly understanding RNAi mechanisms, sequence information and improved algorithms. dsRNA treatment at a high concentration may saturate RNAi core machinery and activate the immune systems, which may cause hazardous effects on the organisms (De Schutter et al., 2022). It is needed to determine the optimal dsRNA concentrations to minimize the potential risk for non-target species. Though the naked dsRNAs have a short half life after foliar spraying or root irrigation, association with nanoparticles and other formulations will prolong the persistence of dsRNA. The potential risks of these additives should be taken into consideration.

### 6.3 dsRNA pesticides regulation

Despite that the sprayable dsRNA pesticides will reach the market soon, a clear regulatory framework has yet to be developed (Rank and Koch, 2021; De Schutter et al., 2022). The Australian Pesticides and Veterinary Medicines Authority (APVMA), US Environmental Protection Agency (EPA) as well as the European Food Safety Authority (EFSA) have utilized the existing regulatory frameworks for the agricultural chemical products, biochemical pesticides and plant protection products as the basis to evaluate dsRNA pesticides, respectively. Additionally, the meeting organized by the Organization for Economic Co-operation and Development (OECD) developed a set of recommendations for risk assessment considerations of the exogenously-applied dsRNA-based products (OECD, 2020). With an upswing market interest in dsRNA-based pesticides, drafting a consensus regulatory framework will facilitate the commercialization process.
