# Supplementary material for: RNAi-based pest control: Production, application and the fate of dsRNA
Source: Front Bioeng Biotechnol. 2022 Nov 29;10:1080576. doi: 10.3389/fbioe.2022.1080576 (PMC9744970; doi:10.3389/fbioe.2022.1080576)
Supplement: Supplementary file 1 [file Table1.DOCX]

**Table S1 dsRNA stability in body fluid and RNases in insects**

| **Order** | **Species** | **dsRNase No.** | **GenBank accession No.** | **Accession number** | **dsRNA degrading activity** | **dsRNase function analysis** | | | | **References** |
| --- | --- | --- | --- | --- | --- | --- | --- | --- | --- | --- |
|  |  |  |  |  |  | **Method** | **dsRNase expression level** | **Target gene dsRNA stability** | **Target gene RNAi response** |  |
| Hymenoptera | Tawny crazy ant  *Nylanderia fulva* | 1 | XM_029315357.1 | XP_029171190.1 | dsGFP degraded 77% after incubation with midgut fluid for 2 h | Feeding | 72 and 61% transcript reduction in the whole body and abdomen | Increased by 42% in midgut fluid | - | (Lei et al., 2022) |
| Dipter | Mosquito  *Aedes aegypti* | 2 | XM_001648419.2  XM_001653429.2 | XP_001648469.1  XP_001653479.2 |  | Feeding | - | Larvae fed with dsRNase showed no significant degradation of dsRNA | Co-feeding shRNA targeting dsRNase and eCFP: RNAi efficiency increased by 24% | (Giesbrecht et al., 2020) |
| Diptera | spotted-wing *drosophila*,  *Drosophila suzukii* | 2 | MW984608  MW984609 | QXY82428.1  QXY82429.1 | The enzyme from 5 guts started to break down the substrate dsRNA and a 10 gut-equivalent seemed to degrade it completely  dsRNase1 was likely more active than dsRNase2 | - | - | - | - | (Yoon et al., 2021) |
| Diptera | Queensland fruit fly  *Bactrocera tryoni* | 2 | JHQJ01007430.1  JHQJ01002120.1 | - | dsRNA incubating with gut extracts showed considerable or complete degradation at 10 and 60 min. | Injection/Feeding | 64-88% transcript reduction | dsRNA exposed to gut extracts injected with dsRNase 1/2 showed no or little degradation. | dsdsRNases Injection/Feeding; Feeding *dsyellow*:  Co-delivery significantly increased the RNAi efficiency; resulting in the complete knockdown of *yellow* transcripts. | (Tayler et al., 2019) |
| Coleopteran | *Tribolium castaneum* | 4 | MN167472.1  MN167473.1  MN167474.1  MN167475.1 | QJD55726.1  QJD55727.1  QJD55728.1  QJD55729.1 | dsRNA degradation rate in the gut tissue was 33.6 times that in the carcass | Injection | >80% transcript reduction | - | *ds-TcdsRNase1* Injection;  *ds-TcLac2a/*TcCYP6BK13 Injection: RNAi efficiency improved by 38.6%-58.9%;  *ds-TcLac2a/*TcCYP6BK13 feeding: RNAi efficiency improved 20.9%-53.9% | (Peng et al., 2020a) |
| Coleopteran | Sweet potato weevil  *Cylas puncticollis* | 3 | MK510881.1  MK510880.1  MK510882.1 | QCF41178.1  QCF41179.1  QCF41177.1 | The integrity of dsGFP was affected after 5 min of incubation with midgut juice, resulting in a smeared  and fainted band | Injection | >70% transcript reduction | dsRNA was degraded  after 15 min of incubating with the gut juice of the dsGFP-injected group.  DsRNA was still stable after 1 h of incubating with the gut juice of the dsdsRN3-injected group. | Injection of *dsdsRN3*, Injection and feeding *dsSnf7*: mortality increased by ~30%; | (Prentice et al., 2017; Prentice et al., 2019) |
| Coleopteran | Colorado potato beetle  *Leptinotarsa decemlineata* | 2 | KX652406.1  KX652407.1 | APF31792.1  APF31793.1 | 1 h incubation with midgut juice: degraded, visible smear band  16 h incubation with midgut juice: completely degraded | Feeding | Transcript levels were reduced by 84% and 86% for *LdsRNAse1* and  *LddsRNase2*, respectively |  | *dsLdarf4* predigested with gut enzymes and then fed to larvae:  1 h predigestion: knockdown efficiency decreased by 28%; 16 h predigestion: no knockdown  feeding *dsdsRNase* then feeding *dsLdarf4/dsLlethtgt*;  RNAi efficiency improved by 11-21%;  Removal of nuclease activity in adults increased the sensitivity towards dsRNA and resulted in improved protection of potato plants. | (Spit et al., 2017) |
| Orthoptera | *Locusta migratoria* | 4 | KY274844.1  KY274845.1  KY386893.1  KY386894.1 | ARW74134.1  ARW74135.1  ARW74136.1  ARW74137.1 | dsRNA is more rapidly degraded in midgut fluid than in hemolymph due to nuclease enzyme activity. | Injection | Significantly reduced the expression | Suppression of *LmdsRNase2* resulted in increased persistence of dsRNA when mixed with  midgut fluid | Oral administration of *dsLmCht10* or *dsLmCHS1* after *dsLmdsRNase2*-injected  Oral administration of dsLmCht10 or dsLmCHS1 after RNAi of LmdsRNase2 caused significant reductions in the transcript levels of *LmCht10* or *LmCHS1,* resulting in 48 and 22% mortality in the *dsLmCht10* and *dsLmCHS1*-treated groups | (Song et al., 2019; Song et al., 2017) |
| Orthoptera | desert locust  *Schistocerca gregaria* | 4 | KJ135008.1  KJ135009.1  KJ135010.1  KJ135011.1 | AHN55088.1  AHN55089.1  AHN55090.1  AHN55091.1 | dsRNA was degraded after incubation with midgut juice | Injection | significantly reduced the expression | Suppression of *dsRNase2* would protect the dsRNA from degrading | - | (Wynant et al., 2014) |
| Hemipteran | pea aphid  *Acyrthosiphon pisum* | 1 | XM_003242604.4 | XP_003242652.1 | dsGFP in the diets was degraded after inoculate with aphids | Feeding | a significant reduction of  nuc1 expression in gut tissues | the 370 nt ds-GFP band was readily  detectable in diet samples supplemented with the ds-nuc1, while the smear of degraded  ds-GFP products were extended to lower molecular weight in the diets  without ds-nuc1. | - | (Chung et al., 2018) |
| hemipteran | whitefly  *Bemisia tabaci* | 2 | KX390872.1  KX390873.1 | AQU43106.1  AQU43107.1 | extra-oral degradation of dsRNA is not substantial  Incubation of dsRNA  with hemolymph for 3 h resulted in its degradation | Feeding | dsRNase1 and dsRNase2 reduced their expression by 25-30% | The intensity of the full-length dsGFP band and ca. 21 nt GFP was elevated in whiteflies administered dsRNA  against the dsRNase gene  When hemolymph  was isolated from dsRNases knockdown whitefly, the incubated dsRNA didn’t show notable degradation of dsRNA till 5 h | Oral administration with dsRNA against dsRNase and SUC1/ AQP1:  of ds-dsRNase1&2 in the diet did not significantly reduce the gene expression level of SUC1/ AQP1  Feeding of dsRNA of the target gene(s) along with dsRNA against dsRNase (dsdsRNase) enhanced the RNAi efficiency by 2.3-31.2% | (Kaur et al., 2020; Luo et al., 2017) |
| hemipteran | Southern Green Stinkbug  *Nezara viridula* | 1 | - |  | In saliva: *dsαCop* in the GFP-injected group was completely degraded after 30min incubation | Injection | a 98% reduction in the transcript | In saliva: started degradation after 10 min incubation; a partial of *dsαCop* in the dsRNase-injected group could be found after 120 min incubation.  In midgut juice: *dsαCop* remained intact for longer in dsRNase-injected adults | dsRNase Injection, feeding dsαCop:  The mortality rate increased by 18.33%  the expression of *αCop* reduced by 48% | (Sharma et al., 2021) |
| Lepidoptera | tobacco hornworm  *Manduca sexta* | 2 | - |  | Smearing and weakening of the dsRNA band began to occur after incubation in hemolymph for just 1 h; after 3 h the dsRNA band had almost entirely disappeared;  dsGFP injected into the larvae disappeared rapidly in hemolymph in vivo | - |  | - | - | (Garbutt et al., 2013) |
| Lepidoptera | *Bombyx mori* | 1 | NM_001098274.1 | NP_001091744.1 |  |  |  |  |  | (Arimatsu et al., 2007) |
| Lepidoptera | tobacco cutworm  *Spodoptera litura* | 5 | MK640212.1  MK640213.1  MK640214.1  MK640215.1  MK640216.1 | QJD55608.1  QJD55609.1  QJD55610.1  QJD55611.1  QJD55612.1 | More active in larvae fed on cabbage leaves as compared to larvae fed on an artificial diet; | CRISPR/Cas9 | - | Knockout of *SldsRNase1, SldsaRNase2* and both of them resulted in 79%, 40% and 96% decrease in dsRNA-degrading activity;  dsRNA-degrading activity in SL1SL2KO  strain showed a decrease of 83.2% in the gut fluid and 23.3%  in hemolymph | SL1SL2KO fed on *dsCYP* showed a 23.2% decrease in *CYP* mRNA levels | (Peng et al., 2020b; Peng et al., 2021) |
| Lepidoptera | diamondback moth  *Plutella xylostella* | 4 | MZ517187.1  MZ517188.1  MZ517189.1  MZ517190.1 | QZW25237.1  QZW25238.1  QZW25239.1  QZW25240.1 | 120 ng dsRNA of was totally degraded by 10 μg of total proteins extracted from the gut or hemolymph in 6 h;  The recombinant dsRNase1 could degrade dsRNA. | Feeding/Injection | Injection: a significant reduction for all *PxdsRNases*  Feeding: a significant reduction for *PxdsRNase1 and 2*, no suppression for *PxdsRNase3 and 4* | - | co-Injection *dsdsRNase & dsCht*: RNAi efficiency was improved after suppression *dsRNases 1 2 3*;  co-feeding *dsRNase* & *dsCht*: RNAi efficiency was improved after suppression *dsRNases 1 2 4* | (Chen et al., 2021) |
| Lepidoptera | European corn borer  *Ostrinia* nubilalis | 4 | MT524715.1  MT524712.1  MT524713.1  MT524714.1 | QOE54913.1  QOE54910.1  QOE54911.1  QOE54912.1 | - | - | - | - | - | (Cooper et al., 2020) |
| Lepidoptera | Asian corn borer,  *Ostrinia furnacalis* | 4 | XM_028302198.1  XP_028158051.1  XM_028306522.1  XM_028302954.1 | XP_028157999.1  XP_028158051.1  XP_028162323.1  XP_028158755.1 | - | - | - | - | In the cultured midgut, suppression of *OfdsRNase2* improved the silencing efficiency of *OfHex1* | (Fan et al., 2021) |

# References

Arimatsu, Y., Kotani, E., Sugimura, Y., Furusawa, T., 2007. Molecular characterization of a cDNA encoding extracellular dsRNase and its expression in the silkworm, Bombyx mori. Insect Biochem Mol Biol 37, 176-183.

Chen, J.Z., Jiang, Y.X., Li, M.W., Li, J.W., Zha, B.H., Yang, G., 2021. Double-Stranded RNA-Degrading Enzymes Reduce the Efficiency of RNA Interference in *Plutella xylostella*. Insects 12.

Chung, S.H., Jing, X., Luo, Y., Douglas, A.E., 2018. Targeting symbiosis-related insect genes by RNAi in the pea aphid-Buchnera symbiosis. Insect Biochem Mol Biol 95, 55-63.

Cooper, A.M.W., Song, H., Shi, X., Yu, Z., Lorenzen, M., Silver, K., Zhang, J., Zhu, K.Y., 2020. Molecular Characterizations of Double-Stranded RNA Degrading Nuclease Genes from Ostrinia nubilalis. Insects 11.

Fan, Y.H., Song, H.F., Abbas, M., Wang, Y.L., Li, T., Ma, E.B., Cooper, A.M.W., Silver, K., Zhu, K.Y., Zhang, J.Z., 2021. A dsRNA-degrading nuclease (dsRNase2) limits RNAi efficiency in the Asian corn borer (Ostrinia furnacalis). Insect Sci 28, 1677-1689.

Garbutt, J.S., Belles, X., Richards, E.H., Reynolds, S.E., 2013. Persistence of double-stranded RNA in insect hemolymph as a potential determiner of RNA interference success: evidence from *Manduca sexta* and *Blattella germanica*. J Insect Physiol 59, 171-178.

Giesbrecht, D., Heschuk, D., Wiens, I., Boguski, D., LaChance, P., Whyard, S., 2020. RNA Interference Is Enhanced by Knockdown of double-stranded RNases in the Yellow Fever Mosquito *Aedes Aegypti*. Insects 11.

Kaur, R., Gupta, M., Singh, S., Joshi, N., Sharma, A., 2020. Enhancing RNAi Efficiency to Decipher the Functional Response of Potential Genes in Bemisia tabaci AsiaII-1 (Gennadius) Through dsRNA Feeding Assays. Front Physiol 11, 123.

Lei, J., Tan, Y., List, F., Puckett, R., Tarone, A.M., Vargo, E.L., Zhu-Salzman, K., 2022. Cloning and Functional Characterization of a Double-Stranded RNA-Degrading Nuclease in the Tawny Crazy Ant (*Nylanderia fulva*). Front Physiol 13, 833652.

Luo, Y., Chen, Q., Luan, J., Chung, S.H., Van Eck, J., Turgeon, R., Douglas, A.E., 2017. Towards an understanding of the molecular basis of effective RNAi against a global insect pest, the whitefly *Bemisia tabaci*. Insect Biochem Mol Biol 88, 21-29.

Peng, Y., Wang, K., Chen, J., Wang, J., Zhang, H., Ze, L., Zhu, G., Zhao, C., Xiao, H., Han, Z., 2020a. Identification of a double-stranded RNA-degrading nuclease influencing both ingestion and Injection RNA interference efficiency in the red flour beetle *Tribolium castaneum*. Insect Biochem Mol Biol 125, 103440.

Peng, Y., Wang, K., Zhu, G., Han, Q., Chen, J., Elzaki, M.E.A., Sheng, C., Zhao, C., Palli, S.R., Han, Z., 2020b. Identification and characterization of multiple dsRNases from a lepidopteran insect, the tobacco cutworm, *Spodoptera litura* (Lepidoptera: Noctuidae). Pestic Biochem Physiol 162, 86-95.

Peng, Y.C., Zhu, G.H., Wang, K.X., Chen, J.S., Liu, X.L., Wu, M., Zhao, C.Q., Xiao, H.J., Palli, S.R., Han, Z.J., 2021. Knockout of SldsRNase1 and SldsRNase2 revealed their function in dsRNA degradation and contribution to RNAi efficiency in the tobacco cutworm, *Spodoptera litura*. J Pest Sci 94, 1449-1460.

Prentice, K., Christiaens, O., Pertry, I., Bailey, A., Niblett, C., Ghislain, M., Gheysen, G., Smagghe, G., 2017. RNAi-based gene silencing through dsRNA Injection or ingestion against the African sweet potato weevil *Cylas puncticollis* (Coleoptera: Brentidae). Pest Manag Sci 73, 44-52.

Prentice, K., Smagghe, G., Gheysen, G., Christiaens, O., 2019. Nuclease activity decreases the RNAi response in the sweetpotato weevil *Cylas puncticollis*. Insect Biochem Mol Biol 110, 80-89.

Sharma, R., Taning, C.N.T., Smagghe, G., Christiaens, O., 2021. Silencing of Double-Stranded Ribonuclease Improves Oral RNAi Efficacy in Southern Green Stinkbug *Nezara viridula*. Insects 12.

Song, H., Fan, Y., Zhang, J., Cooper, A.M., Silver, K., Li, D., Li, T., Ma, E., Zhu, K.Y., Zhang, J., 2019. Contributions of dsRNases to differential RNAi efficiencies between the Injection and oral delivery of dsRNA in *Locusta migratoria*. Pest Manag Sci 75, 1707-1717.

Song, H., Zhang, J., Li, D., Cooper, A.M.W., Silver, K., Li, T., Liu, X., Ma, E., Zhu, K.Y., Zhang, J., 2017. A double-stranded RNA degrading enzyme reduces the efficiency of oral RNA interference in migratory locust. Insect Biochem Mol Biol 86, 68-80.

Spit, J., Philips, A., Wynant, N., Santos, D., Plaetinck, G., Vanden Broeck, J., 2017. Knockdown of nuclease activity in the gut enhances RNAi efficiency in the Colorado potato beetle, *Leptinotarsa decemlineata*, but not in the desert locust, *Schistocerca gregaria*. Insect Biochem Mol Biol 81, 103-116.

Tayler, A., Heschuk, D., Giesbrecht, D., Park, J.Y., Whyard, S., 2019. Efficiency of RNA interference is improved by knockdown of dsRNA nucleases in tephritid fruit flies. Open Biol 9, 190198.

Wynant, N., Santos, D., Verdonck, R., Spit, J., Van Wielendaele, P., Vanden Broeck, J., 2014. Identification, functional characterization and phylogenetic analysis of double stranded RNA degrading enzymes present in the gut of the desert locust, *Schistocerca gregaria*. Insect Biochem Mol Biol 46, 1-8.

Yoon, J.S., Ahn, S.J., Flinn, C.M., Choi, M.Y., 2021. Identification and functional analysis of dsRNases in spotted-wing drosophila, *Drosophila suzukii*. Arch Insect Biochem Physiol 107, e21822.
